# Retention and Distribution of Dopamine-Dependent Reward Memory in Regenerating Planaria

**DOI:** 10.3390/biom16050649

**Published:** 2026-04-27

**Authors:** Kenneth Samuel, Abigail K. Hakes, Easter S. Suviseshamuthu, Maria E. Fichera

**Affiliations:** 1 Department of Molecular and Integrative Physiology, University of Illinois Urbana-Champaign, Urbana, IL 61801, USA; 2 Department of Biology, Eastern University, St. Davids, PA 19087, USA; abigail.hakes@eastern.edu (A.K.H.); mfichera@eastern.edu (M.E.F.); 3 Center for Mobility and Rehabilitation Engineering Research, Kessler Foundation, West Orange, NJ 07052, USA

**Keywords:** addiction-like behavior, conditioned place preference, D1 dopamine antagonist, memory retention, planaria

## Abstract

Memory is generally thought to be stored within centralized neural circuits. However, whether learned behaviors can persist in the absence of a brain remains unresolved. Planaria (*Girardia* spp.) possess a primitive cephalic ganglion and a remarkable capacity for regeneration, providing a unique system to examine non-cephalic memory retention. The primary aim of this study was to determine whether sucrose-induced conditioned place preference (CPP) is retained in posterior, brainless planarian fragments. Planaria were trained using a Pavlovian conditioning paradigm in which an initially unpreferred surface was paired with a 10% sucrose solution, resulting in a robust shift in surface preference. Following amputation, anterior fragments containing the cephalic ganglion as well as posterior fragments lacking the brain preserved the conditioned preference, demonstrating that reward-associated memory is stored even outside the cephalic nervous system. As a secondary objective, we examined the role of dopaminergic reinforcement using a D1 dopamine receptor antagonist during training. While antagonist-treated planaria failed to develop a CPP, posterior fragments from these amputated planaria likewise showed no conditioned preference, indicating that dopamine-dependent signaling is essential for sucrose-associated memory formation across the body. These results provide support for the hypothesis that reward-associated memory in planaria is distributed beyond the brain and can be modulated by dopaminergic pathways, highlighting the utility of this model for exploring fundamental mechanisms of reward, memory, and potential pharmacological interventions.

## 1. Introduction

Planaria are flatworms belonging to the phylum *Platyhelminthes*, class *Rhabditophora*, and order *Tricladida* [1]. These invertebrates are bilaterally symmetrical, triploblastic, and acoelomate, and are commonly found in freshwater habitats such as ponds, lakes, and rivers, although some species inhabit saltwater or terrestrial environments [2,3]. Planaria exhibit distinct behavioral preferences, including sensitivity to light, electric and magnetic fields, chemical gradients, and surface textures, typically favoring rough over smooth surfaces [4,5,6,7]. These inherent behavioral tendencies make planaria an ideal system for investigating how environmental cues shape behavior and for exploring the neural mechanisms underlying learning and memory.

A defining feature of planaria is their extraordinary regenerative capacity. They reproduce asexually via binary fission, and any fragment of a planarian can regenerate into a complete, fully functional organism [2,8,9,10,11,12,13,14]. This remarkable ability is primarily attributed to a high concentration of pluripotent stem cells, called neoblasts, which constitute roughly 25% of the organism [11,13,15]. In addition to their regenerative properties, planaria possess a primitive “brain” located in the anterior region, within the cephalic ganglion, which contains specialized sensory structures and nerve cells [1,7,16]. Despite its simplicity, the planarian nervous system exhibits many structural and neurochemical features similar to vertebrates, including the presence of nearly all mammalian neurotransmitters, such as dopamine, and comparable receptor systems [17,18,19]. These similarities allow researchers to investigate fundamental neural processes in a simple organism while maintaining relevance to more complex species.

Planaria are particularly well suited for the present study because of their remarkable ability to regenerate missing body structures, including the head and nervous system, after amputation. This feature makes them an especially appropriate model for investigating whether reward-associated memory can persist in body fragments following loss of the cephalic ganglion. In addition, their responses to environmental and chemical stimuli can be observed directly, supporting their use in behavioral studies of drug-induced effects, toxicology, and neuropharmacology [17]. Thus, in the context of the present work, the choice of planaria is motivated primarily by their biological suitability for examining memory retention across regeneration.

Planaria have been widely employed in studies of drug-induced behavioral changes and addiction-like phenomena [20,21]. Exposure to psychoactive substances—including cocaine, nicotine, opioids, amphetamines, cannabinoids, and sucrose—can elicit conditioned behaviors that parallel addiction-related responses observed in vertebrates, including withdrawal symptoms [21,22,23]. In humans, genetic and epigenetic factors substantially contribute to addiction vulnerability, with approximately 40–60% of risk estimated to be heritable [24]. For instance, children of alcohol abusers are three- to five-fold more likely to develop addictive behaviors than children of non-alcoholic parents [24]. These findings highlight the relevance of conserved biological mechanisms across species. Because planaria possess neurotransmitter systems and neural circuits analogous to those in mammals, they provide a valuable model for investigating the fundamental processes underlying addiction-like behavior and reward [19,25].

Sucrose, a naturally rewarding substance, has been shown to induce addiction-like behavior in planaria, with neurochemical responses resembling those elicited by drugs of abuse [23]. Repeated exposure to a sucrose solution can establish a conditioned place preference (CPP), in which planaria associate a previously non-preferred surface with a rewarding stimulus [6]. This Pavlovian conditioning paradigm provides a window into associative learning, demonstrating how environmental cues become linked with rewarding experiences. Planaria are also capable of acquiring long-term memory, which can persist even after amputation and subsequent brain regeneration [4,26,27]. Some studies have proposed that memory can be transferred through cannibalism or RNA-mediated mechanisms, although these claims remain debated [28,29,30]. Collectively, these observations raise an important question: is reward-associated memory confined to the cephalic ganglion, or can it also be stored and expressed in posterior, brainless segments following amputation?

Primary Aim: The main objective of this study was to investigate whether sucrose-induced reward-associated memory is retained in both anterior (head-containing) and posterior (brainless) segments of planaria following amputation. If addiction-like behavior involves non-cephalic or distributed memory mechanisms, the posterior fragment may retain the learned preference even in the absence of the cephalic ganglion. We hypothesized that the brainless posterior fragment would preserve reward-associated memory and exhibit a CPP comparable to that of the anterior segment.

Secondary Aim: We also investigated whether disrupting dopaminergic signaling with a D1 dopamine receptor antagonist could prevent the acquisition of a sucrose-induced CPP [19,25]. Importantly, we tested whether D1 antagonism is sufficient to block reward-associated memory formation in both anterior and posterior segments, representing a novel aspect of this study. We hypothesized that planaria exposed to a D1 antagonist would fail to develop a CPP, indicating that dopamine-dependent reinforcement is necessary for the formation of sucrose-associated memory across both fragments.

Approach: To address these objectives, we first verified the innate surface preferences of intact and fragmented planaria. Next, we trained planaria in a CPP paradigm using a sucrose solution paired with a previously non-preferred surface. Following amputation, we assessed whether posterior fragments retained the conditioned preference. Finally, we evaluated the effect of D1 antagonism during CPP training in both intact and amputated fragments. This experimental design allows for an examination of the segmental requirements for the retention of addiction-like behavior and the identification of dopaminergic signaling in reward-associated learning.

Taken together, these studies demonstrate that planaria provide a powerful model for examining distributed memory storage, addiction-like behavior, and dopaminergic modulation. By investigating how memory and addiction-like behavior persist outside of the cephalic ganglion, this work aims to provide novel insights into the molecular and cellular mechanisms underlying associative learning and reward across species, highlighting the broader significance of simple invertebrate models in understanding complex neurobiological processes.

## 2. Materials and Methods

### 2.1. Subjects

**Figure 1:**
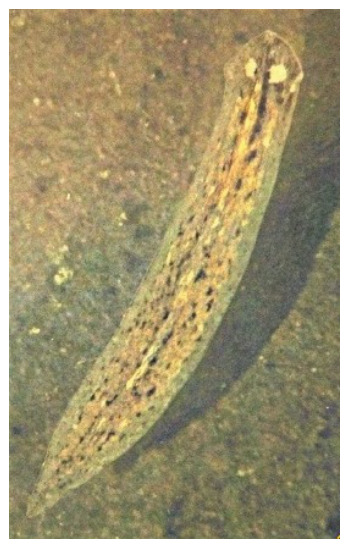
A specimen of planaria belonging to the genus *Girardia* investigated in this study. Figure courtesy of [31].

We performed the experiments with planaria specimens that belong to the *Girardia* genus shown in Figure 1. The planaria were kept in a bowl containing Evian natural spring water filled up to 1 cm, at room temperature. They were fed egg yolk before the commencement of the experiment. Feeding was stopped a day before starting the training period.

### 2.2. Materials

**Figure 2:**
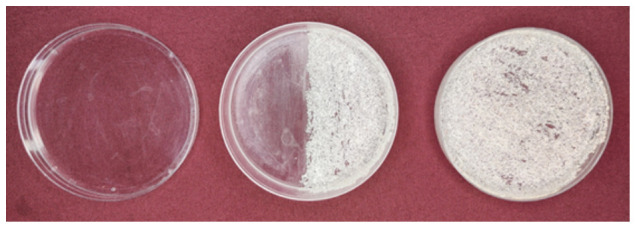
Training phase Petri dishes. (**Left**): smooth surface (initially unpreferred), paired with sucrose solution. (**Middle**): half-rough dish, divided into rough and smooth halves to be used for the pre-and post-training tests. (**Right**): rough surface (initially preferred), paired with water.

The experiments were conducted with three Petri dishes of 9 cm diameter. One Petri dish was kept unaltered to serve as a smooth surface for the planaria to glide on (Figure 2, left). White sand was stuck to the inner and bottom surface of the second dish to make it rough (Figure 2, right). A transparent silicone adhesive (DAP 00688 all-purpose adhesive sealant, 100% silicone, 2.8-ounce tube) was used for this purpose, which took about 72 h to dry completely and cure properly. The third Petri dish was divided into two sections as follows: one half was left smooth while the other half was glued with sand (hereafter referred to as half-rough dish, middle dish shown in Figure 2).

We prepared a 100 mL solution containing 10% sucrose for training the planaria. A D1 dopamine antagonist (SCH-23390 hydrochloride, Sigma Aldrich, St. Louis, MO, USA) was obtained to test the secondary hypothesis. A 10 mM stock solution of SCH-23390 was prepared in DMSO and stored at 4 °C throughout the 12-day training period. From this stock, a 1 μM SCH-23390 solution was prepared in 10% sucrose, stored at 4 °C, and used daily during the 12-day training period. The selected dose was based on the study by Zhang et al. [23], who used SCH-23390 at 1 μM and reported that the development of sucrose-induced CPP in planaria was inhibited in the presence of this dopamine receptor antagonist.

### 2.3. Methods

**Figure 3:**
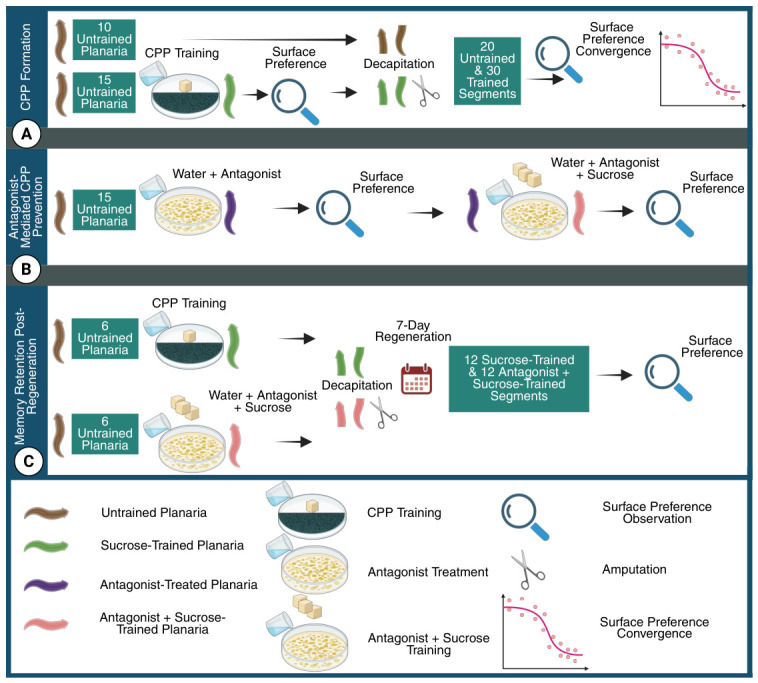
The study consisted of the following three experimental stages. A In the first stage, the baseline surface preference of planaria (n=15) was recorded and subsequently reassessed following training with a 10% sucrose solution to induce CPP. The sucrose-trained animals (n=15) and an additional cohort of untrained planaria (n=10) were then amputated into head and tail segments, yielding 30 segments from trained animals and 20 from untrained controls. The rate of convergence of segments from each cohort toward their respective preferred surfaces was subsequently compared. B In the second stage, a single cohort of planaria (n=15) was used. The animals were first introduced to the antagonist alone, and their surface preference was recorded to establish a control condition. The same planaria were subsequently trained with both the antagonist and a 10% sucrose solution, after which their surface preference was reassessed. The resulting preference was compared with that observed under the antagonist-only condition to evaluate the antagonist’s ability to prevent CPP formation. C In the third stage, two additional cohorts (n=6 each) were trained with either 10% sucrose solution alone or with sucrose and the antagonist. Animals from each cohort were amputated to generate 12 segments (six heads and six tails), which were allowed to regenerate for seven days. CPP retention in the sucrose-trained cohort and antagonist-mediated prevention of CPP in the sucrose-plus-antagonist cohort were subsequently assessed following regeneration.

As illustrated schematically in Figure 3, the study was conducted using a pool of 52 planaria, and it comprised three experimental stages: (i) CPP formation; (ii) antagonist-mediated prevention of CPP; and (iii) evaluation of reward-associated memory (CPP) retention following regeneration. In the sequel, a detailed account on each stage is provided.

Behavioral experiments were conducted by one investigator, whereas outcome assessment and statistical analysis were carried out independently by a second investigator who was blinded to the study hypothesis, thereby reducing the risk of observer and analytical bias.

#### 2.3.1. Conditioned Place Preference Formation

**Establishment of CPP**. The experiment was divided into the following three phases.

Phase 1—Pre-training Phase: On the first day, a cohort of 15 untrained planaria were individually transferred to the half-rough dish for 30 min. The time spent by each planaria on either surface (smooth and rough) was recorded. The surface preferred by a planaria was determined to be the one where it spent a longer time. In other words, the duration spent on a surface was considered to be the indicator of the level of preference.

Phase 2—Training Phase: The preferred surface was paired with water (P+W) and the unpreferred surface with 10% sucrose solution (U+S). To reduce potential bias arising from sequence effects, exposure to P+W and U+S was not administered in a fixed order; instead, the order was varied across training sessions in a mostly random manner, rather than consistently presenting the sucrose-paired condition later. The use of sucrose as a rewarding stimulus was supported by earlier work showing that sucrose produces reinforcing effects in planaria [23]. For the present study, a 10% sucrose solution was chosen based on evidence from Mohammed et al. [6], who demonstrated that planarians exposed to 10% sucrose developed a CPP that could be extinguished and reinstated, indicating that this concentration is sufficient to support robust associative reward learning. The training phase was designed to last for 12 days. During this period, the planaria were transferred from the P+W to the U+S surface for 30 min each day. Of note, a training paradigm was initially attempted, wherein a cohort of 15 planaria was alternated between the P+W and U+S surface every 24 h for 12 days. However, it failed to produce a significant CPP as observed in other studies [21,23,32]. A plausible explanation is that prolonged exposure to the rewarding stimulus diminished the effectiveness of conditioning, potentially as a consequence of withdrawal-related disruption upon removal from the sucrose environment, as discussed in Section 4.3.

Phase 3—Post-Training Phase: On the 13th day, the sucrose-trained planaria were tested to see if their surface preference had altered. Each animal was placed on the half-rough Petri dish for 30 min and the respective time spent on the rough and smooth surface was noted.

**Surface Preference Convergence Rate**. After establishing CPP, the 15 sucrose-trained planaria were amputated transversely to generate head and tail fragments 24 h after the final training session [4] and placed on the unpreferred surface of the half-rough dish. The number of anterior (head) and posterior (tail) segments on the rough and smooth surface was counted every minute for 30 min.

Likewise, another cohort of 10 untrained planaria were amputated into head and tail segments and placed on the unpreferred surface of the half-rough dish for 30 min. The number of segments on each surface was recorded every minute.

#### 2.3.2. D1 Dopamine Antagonist-Mediated CPP Prevention

A cohort of 15 untrained planaria was placed on the half-rough dish that contained 1 μM of D1 dopamine antagonist along with regular spring water. Their surface preference was investigated by recording the time spent by each animal on the rough and smooth surface for 30 min.

Next, 1 μM of D1 dopamine antagonist was added to 10% sucrose solution, which was paired with the unpreferred surface (U+S+D). Likewise, the preferred surface was paired with water (P+W). To reduce potential bias arising from sequence effects, exposure to P+W and U+S+D was not administered in a fixed order; instead, the order was varied across training sessions in a mostly random manner. The training phase consisted of transferring planaria from the P+W surface to the U+S+D surface for 30 min each day, for 12 days. On the 13th day, the antagonist-mediated CPP prevention was verified by observing the surface preference of each sucrose + antagonist-trained planaria on the half-rough dish for 30 min.

#### 2.3.3. Memory Retention Post-Regeneration

A cohort of six sucrose-trained and another cohort of six sucrose+antagonist-trained planaria were amputated transversely into head and tail segments 24 h after the final training session [4]. The segments were allowed to regenerate for seven days since planaria exhibit remarkable regenerative abilities, capable of completely regenerating missing structures, including the nervous system, within less than seven days following amputation [33]. In each cohort, the surface preference of the 12 animals—regenerated from the anterior and posterior segments—was examined by placing them on the half-rough dish for 30 min and recording the time spent by each regenerated planaria on both surfaces.

## 3. Results

The preliminary results of this study were presented in [34].

### 3.1. CPP Formation

#### 3.1.1. Untrained Planaria

**Figure 4:**
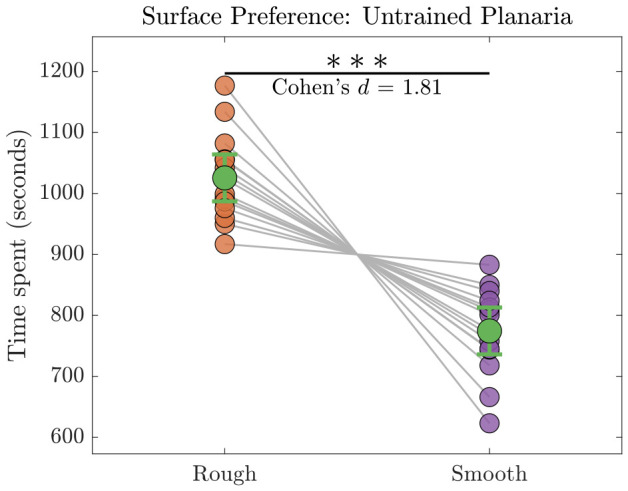
Surface preference of untrained planaria. Time spent by untrained planaria on rough versus smooth surfaces (n=15). Each line connects measurements from a single individual. Scatter points represent individual values, while larger green markers indicate the mean ± 95% confidence interval. The horizontal bar indicates a significant difference between conditions ($p_{\mathrm{BH}}<0.001$), and the associated effect size (Cohen’s d=1.81) is shown above the bar. A statistically significant difference at p<0.001 is denoted by three asterisks (∗∗∗).

This experiment was performed to identify the preferred surface of the untrained planaria. They spent more time on the rough surface (mean ± SEM: 1026 ± 18 s) compared to the smooth surface (mean ± SEM: 774 ± 18 s). A paired comparison of untrained planaria (n=15) surface preference revealed a significant difference in the time spent on the rough versus smooth surfaces. Individual data points are shown with connecting lines, and group means are represented with sage green markers and 95% confidence interval in Figure 4. After verifying the normality of data with Shapiro–Wilk test, a paired *t*-test confirmed this difference (*t*(14) = 7.00, $p<6.257\times 10^{-6}$), with a large effect size (Cohen’s *d* = 1.81) and high post-hoc power (1.00). The asterisk above the bars indicates statistical significance. This visualization highlights the strong preference of untrained planaria for the rough surface.

#### 3.1.2. Sucrose-Trained Planaria

**Figure 5:**
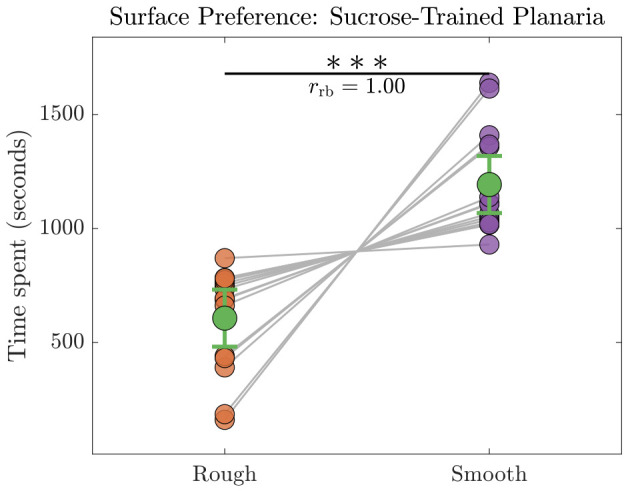
Time spent by sucrose-trained planaria on the rough vs. smooth surfaces (n=15). Individual data points are shown with connecting lines, and group means are represented with green markers and 95% confidence intervals. A Wilcoxon signed-rank test revealed a significant preference ($p_{\mathrm{BH}}<0.001$) for the smooth surface with a maximal effect size ($r_{\mathrm{rb}}=1.00$). Three asterisks (∗∗∗) denote a statistically significant difference at p<0.001.

The sucrose-trained planaria spent an average of 607 s on the rough surface and 1193 s on the smooth surface. This means that their surface preference had shifted to the smooth surface owing to CPP formation. Because the paired samples did not satisfy the assumption of normality as reported by Shapiro–Wilk test, a Wilcoxon signed-rank test was used to compare the time spent on rough versus smooth surfaces (n=15) as shown in Figure 5. The test revealed a significant preference for the smooth surface (W=0, $p=6.10\times 10^{-5}$), with a maximal effect size given by the rank-biserial correlation ($r_{\mathrm{rb}}=1.00$). The Hodges–Lehmann median difference was −414 s, with an exact 95% confidence interval of [−627,−414] s (rough minus smooth).

After applying the Benjamini-Hochberg (BH) correction [35] for multiple comparisons, the adjusted *p*-values were:

$$p_{\mathrm{BH}}^{\mathrm{Untrained}}=6.10\times 10^{-5},\;p_{\mathrm{BH}}^{\mathrm{Sucrose}-\mathrm{Trained}}=1.25\times 10^{-5}.$$

#### 3.1.3. Surface Convergence Rate: Amputated Planaria Segments

**Figure 6:**
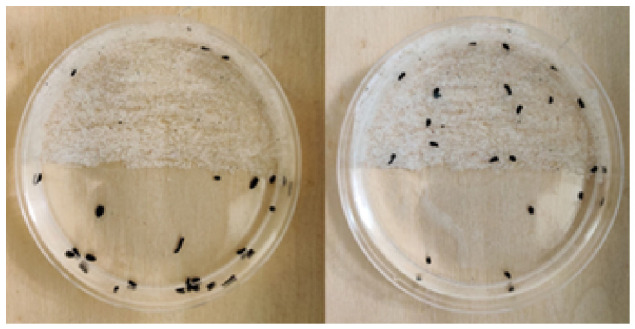
(**Left**): Segments of the sucrose-trained planaria on the half-rough dish after 30 min. Both the anterior and posterior segments show a strong preference for the smooth surface. (**Right**): Segments of the untrained planaria on the half-rough dish after 30 min. The anterior as well as the posterior fragments prefer the rough surface.

**Figure 7:**
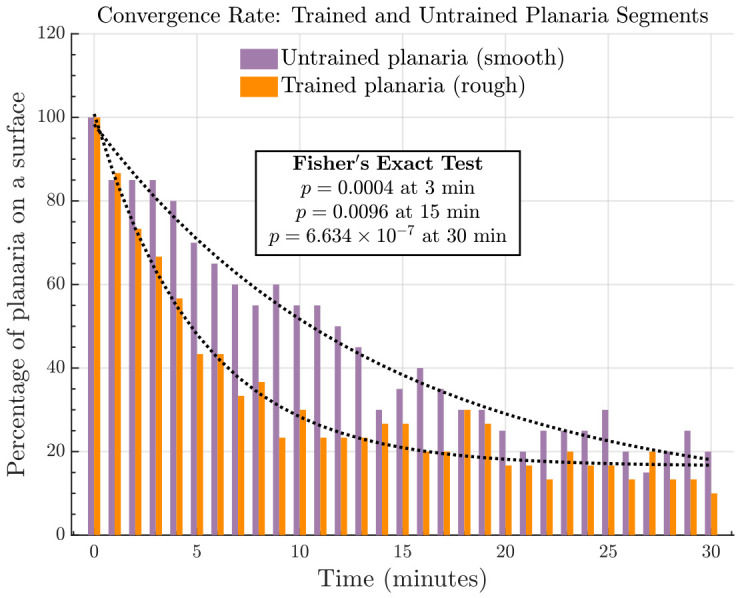
The switching of amputated planaria segments between the rough and smooth surface, which is modeled by the exponential decay curves. The percentage of trained planaria segments present on the unpreferred rough surface is shown in orange and that of the untrained segments on the smooth (unpreferred) surface in purple. Both the groups tend to migrate toward the preferred surface gradually over 30 min. The trained planaria segments that were conditioned by sucrose exhibited a higher rate of transition toward the smooth surface, which might be associated with addiction-like behavior.

The sucrose-trained planaria segments (both head and tail) began to move toward the smooth surface at a faster rate, when they were placed on the rough surface of the half-rough dish. By the end of 30 min, 27 segments had transitioned to the smooth surface while only three segments remained on the rough surface as shown in Figure 6 (left). In contrast, the untrained segments gradually moved toward the preferred rough surface. After 30 min, 16 segments were present on the rough surface and the remaining four on the smooth surface as shown in Figure 6 (right). The transitions were mathematically modeled by the exponential decay curves in Figure 7. Nonlinear regression was performed in MATLAB R2025a using the fitnlm function from the Statistics and Machine Learning Toolbox (MathWorks, Natick, MA, USA) [36], which estimated model parameters by minimizing the sum of squared residuals. The slope of the dotted curve representing the transition rate of sucrose-trained planaria is 0.20, whereas the slope of the curve denoting the transition rate of the untrained planaria is 0.07. Importantly, the rate at which the transition took place toward the preferred surface is higher in the case of sucrose-trained planaria compared to the untrained planaria.

For the dotted curve modeled with the number of untrained planaria segments on the smooth surface (n=20), the estimated parameters were $b_{1}=90.64\pm 4.20$, $b_{2}=0.0722\pm 0.0097$, and $b_{3}=7.66\pm 4.91$ (mean ± standard error). The model exhibited excellent fit ($R^{2}=0.968$, adjusted $R^{2}=0.965$, root mean squared error (RMSE) = 4.48) and was highly significant against a constant model ($F_{1,28}=418$, $p=1.41\times 10^{-21}$). All parameter estimates were statistically significant except $b_{3}$.

For the dotted curve modeled with the number of sucrose-trained planaria segments on the rough surface (n=30), parameter estimates were $b_{1}=84.30\pm 3.16$, $b_{2}=0.197\pm 0.015$, and $b_{3}=16.52\pm 1.26$, with similarly strong fit ($R^{2}=0.967$, adjusted $R^{2}=0.965$, RMSE = 4.24) and *F*-test against a constant model ($F_{1,28}=410$, $p=1.85\times 10^{-21}$). All parameters were statistically significant.

To determine whether the CPP training altered surface preference in amputated planaria segments (head/tail), we compared the proportion of time spent on the smooth versus rough surface between sucrose-trained and untrained segments at 3, 15, and 30 min. Statistical significance was assessed using Fisher’s exact test for 2 × 2 contingency tables at each time point.

As shown in Table 1, sucrose-trained planaria segments exhibited a significant shift in surface preference compared to untrained segments at all three time points (3 min: p=0.0004; 15 min: p=0.0096; 30 min: $p=6.634\times 10^{-7}$), spending more time on the smooth surface after training. For completeness, a more detailed region-wise analysis of surface preference immediately after amputation, including separate head and tail comparisons for untrained and sucrose-trained planaria, is presented in Appendix A.

### 3.2. Antagonist-Mediated CPP Prevention

#### 3.2.1. Antagonist-Treated Planaria

**Figure 8:**
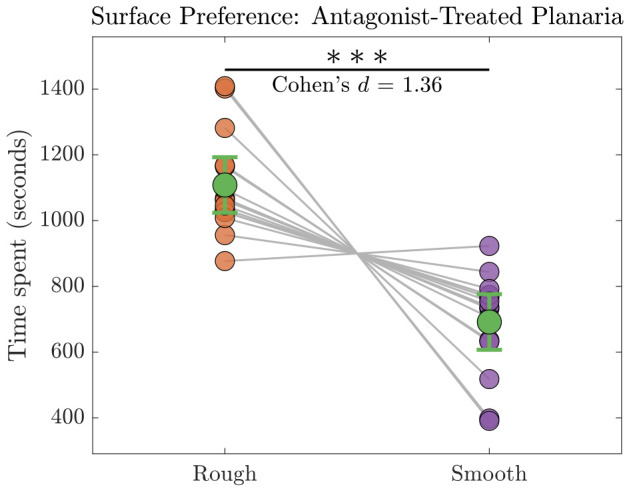
Surface preference of antagonist-treated planaria measured by the time spent by them on rough versus smooth surfaces (n=15). Scatter points represent individual values, and each line connects measurements from an individual. Larger green markers indicate the mean ± 95% confidence interval. The horizontal bar denotes a significant difference between conditions ($p_{\mathrm{BH}}<0.001$), and the associated effect size (Cohen’s d=1.36) is marked above the bar. Statistical significance at p<0.001 is indicated by ∗∗∗.

The objective of the experiment was to identify the surface preference of 15 untrained planaria when introduced to 1 μM of D1 dopamine antagonist. They spent an average of 1109 s on the rough surface and 692 s on the smooth surface. This confirms that the untrained planaria maintained their preference for the rough surface in the presence of D1 antagonist. After verifying normality of data with Shapiro–Wilk test, a paired two-tailed *t*-test was performed, which revealed a significant difference in the preference of antagonist-treated planaria (n=15) between the rough and smooth surfaces (Figure 8). The mean difference was 416.7s (95% CI, 247.4–585.9s), indicating substantially higher values for the rough surface relative to the smooth surface. This effect was statistically significant (t(14)=5.28, $p=1.16\times 10^{-4}$). The magnitude of the effect was large (Cohen’s d=1.36). Post-hoc power analysis indicated high statistical power (1.00), suggesting that the sample size was sufficient to reliably detect the observed effect.

#### 3.2.2. Sucrose + Antagonist-Trained Planaria

**Figure 9:**
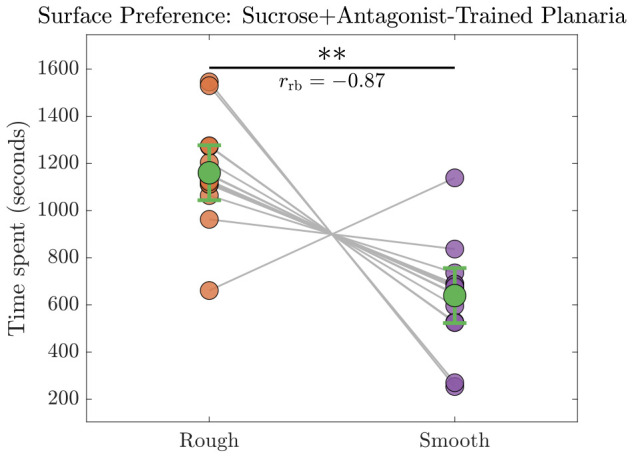
Time spent by sucrose+antagonist-trained planaria on the rough vs. smooth surfaces (n=15). Individual data points are represented with connected lines, and group means are denoted with green markers and 95% confidence intervals. A Wilcoxon signed-rank test revealed a significant preference ($p_{\mathrm{BH}}<0.01$) for the rough surface with an effect size ($r_{\mathrm{rb}}=-0.87$). Statistical significance at p<0.01 is denoted by ∗∗.

Fourteen out of 15 sucrose+antagonist-trained planaria did not switch their preference as can be seen from Figure 9, which confirms that they continued to prefer the rough surface except one. Consequently, the time spent on the rough surface by the planaria trained with 10% sucrose solution and D1 dopamine antagonist remained significantly longer compared to that on the smooth surface. Since the data violated normality assumption as determined by Shapiro–Wilk test, a Wilcoxon signed-rank test was performed to evaluate the difference between the two paired conditions—preference of sucrose + antagonist-trained planaria between the rough and smooth surfaces (Figure 9). The analysis revealed a statistically significant difference (n=15, W=112.00, $p=1.53\times 10^{-3}$), indicating a systematic shift between the paired observations. The Hodges–Lehmann estimator indicated a median difference of 454.00 s, with an exact 95% confidence interval of [454.00,539.00] s. The magnitude of the effect was large, as reflected by the rank-biserial correlation ($r_{\mathrm{rb}}=-0.867$), suggesting a strong directional difference between the paired samples.

After applying the BH correction for multiple comparisons, the adjusted *p*-values were given by:

$$p_{\mathrm{BH}}^{\mathrm{Antagonist}-\mathrm{Treated}}=2.32\times 10^{-4},\;p_{\mathrm{BH}}^{\mathrm{Sucrose}+\mathrm{Antagonist}-\mathrm{Trained}}=1.53\times 10^{-3}.$$

### 3.3. Memory Retention Post-Regeneration

#### 3.3.1. Sucrose-Trained Regenerated Planaria

**Figure 10:**
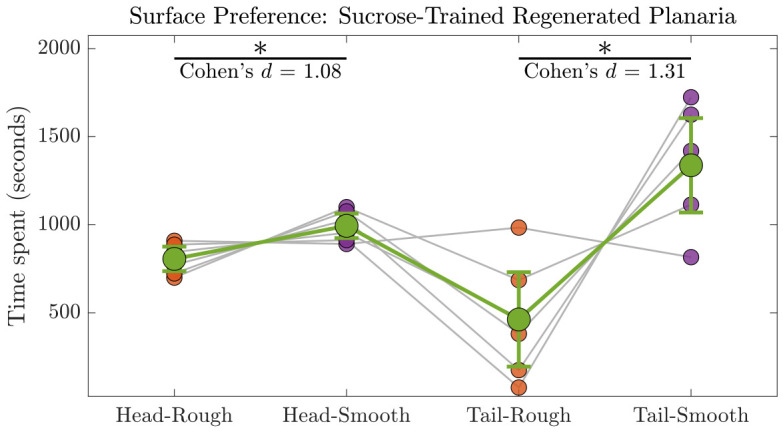
Two-factor RM analysis of time spent by sucrose-trained planaria (n=6) on different surfaces post regeneration. Individual gray lines represent the trajectories of individual planaria across the four conditions: Head-Smooth, Head-Rough, Tail-Smooth, and Tail-Rough. The green dots indicate mean time spent for each condition, with 95% confidence intervals shown as error bars. Green lines connect the means to highlight the overall trend across conditions. Post-hoc paired *t*-tests (BH-corrected) revealed that the planaria regenerated from the head ($p_{\mathrm{BH}}=0.0458,\;d=1.08$) and tail ($p_{\mathrm{BH}}=0.0480,\;d=1.31$) regions spent significantly more time on the smooth surface compared to the rough surface. Statistical significance at p<0.05 is indicated by *.

We tested whether the planaria regenerated from the head/tail regions spent different amounts of time on the rough versus smooth surface using a two-factor repeated-measures (RM) ANOVA, with body region (head vs. tail) and surface type (rough vs. smooth) as within-subject factors (n=6) as shown in Figure 10. Most of the sucrose-trained segments after regeneration maintained their conditioned preference for the smooth surface. Normality of each condition was confirmed with the Shapiro–Wilk test prior to analysis, allowing the use of parametric tests.

The ANOVA revealed a significant main effect of surface on time spent ($\mathrm{surface}:\;F_{1,5}=13.45,\;p=0.0145,\;\eta_{p}^{2}=0.749$), indicating that planaria spent more time on the smooth surface than the rough surface. There was no significant effect of region (head vs. tail: $F_{1,5}=-2.13\times 10^{-13},\;p=1.0,\;\eta_{p}^{2}\approx 0$) and the region × surface interaction was marginally non-significant ($F_{1,5}=6.25,\;p=0.0546,\;\eta_{p}^{2}=0.555$), suggesting that the effect of surface was largely consistent across body regions.

Post-hoc paired *t*-tests with BH correction confirmed that planaria spent significantly more time on the smooth surface regenerated from both the head region ($t_{5}=2.643,\;$$p_{\mathrm{BH}}=0.0458,\;$d=1.08) and the tail region ($t_{5}=3.200,\;p_{\mathrm{BH}}=0.0480,\;d=1.31$), demonstrating large effect sizes for these comparisons.

Overall, these results indicate that planaria have a strong preference for the smooth surface, independent of body region from where they were regenerated, with robust effects confirmed by both the ANOVA and post-hoc tests (refer to Table 2 and Table 3).

#### 3.3.2. Sucrose + Antagonist-Trained Regenerated Planaria

**Figure 11:**
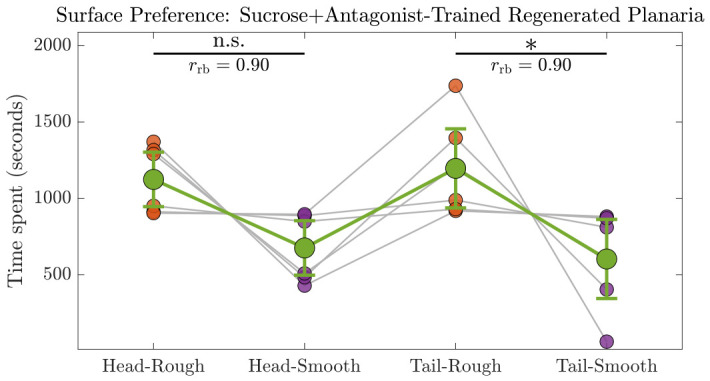
Nonparametric two-factor RM analysis of time spent by sucrose + antagonist-trained planaria (n=6) on the rough and smooth surfaces post regeneration. Gray lines represent the trajectories of individual planaria across the four conditions: Head–Smooth, Head–Rough, Tail–Smooth, and Tail–Rough. Green dots denote the mean time spent in each condition, with 95% confidence intervals shown as error bars. Green lines connect the means to illustrate the general pattern across conditions. Post-hoc Wilcoxon signed-rank tests (BH-corrected) revealed that the planaria regenerated from the head ($p_{\mathrm{BH}}=0.0625$, $r_{\mathrm{rb}}=0.899$) and tail ($p_{\mathrm{BH}}=0.0312$, $r_{\mathrm{rb}}=0.899$) regions spent more time on the rough surface compared to the smooth surface. Statistical significance at p<0.05 is indicated by ∗, while n.s. denotes not significant.

Most of the sucrose + antagonist-trained head and tail segments after regeneration maintained their natural preference for the rough surface as shown in Figure 11. Normality of the time spent on each surface was assessed using the Shapiro–Wilk test, and at least one condition violated the assumption of normality. Therefore, we used a nonparametric two-factor RM design (Scheirer–Ray–Hare test) appropriate for non-normal RM data (n=6) as shown in Figure 11. Surface had a significant effect on time spent ($\eta_{H}^{2}=1.0$, p=0.0011), whereas body region (head vs. tail) and the region × surface interaction were not significant ($\eta_{H}^{2}<0.001$, p>0.6), indicating that planaria spent similar amounts of time irrespective of whether they were regenerated from the head or tail region and that the effect of surface was consistent across regions as summarized in Table 4.

Post-hoc paired Wilcoxon signed-rank tests with BH correction confirmed that planaria spent more time on the smooth surface compared to the rough surface. Specifically, for the head region, the difference was marginally non-significant after correction ($r_{\mathrm{rb}}=0.899$, $p_{\mathrm{BH}}=0.0625$), while for the tail region, the difference was significant ($r_{\mathrm{rb}}=0.899$, $p_{\mathrm{BH}}=0.0312$). These results demonstrate a robust preference for the smooth surface that was consistent across body regions.

### 3.4. A Cautionary Note

Note that the correspondence between individual planaria across experimental conditions—untrained vs. sucrose-trained in Section 3.1 and antagonist-treated vs. sucrose+antagonist-trained in Section 3.2—could not be reconstructed. Therefore, the subject-level structure required for a two-factor RM ANOVA or its nonparametric equivalent was unavailable. RM models require multiple observations obtained from the same experimental unit across conditions in order to account for within-subject correlations [37]. Consequently, comparisons of surface preference (rough vs. smooth) within each training condition were evaluated using a paired two-tailed *t*-test or Wilcoxon signed-rank test based on the data distribution. Since two hypothesis tests were performed, the resulting *p*-values were adjusted using the BH false discovery rate procedure to control for multiple comparisons [35]. Importantly, when a within-subject factor has only two levels, the statistical test performed by RM ANOVA is mathematically equivalent to a paired *t*-test because the ANOVA *F* statistic reduces to the square of the corresponding *t* statistic [38]. Therefore, statistically significant differences identified by the paired *t*-tests or Wilcoxon signed-rank tests are expected to remain significant under the equivalent RM ANOVA framework if full pairing information were available. Nevertheless, because the absence of subject-level correspondence prevented construction of the full RM design matrix, interaction effects between training condition and surface type could not be formally tested, and the results should therefore be interpreted as within-condition comparisons rather than a complete factorial RM analysis.

## 4. Discussions

### 4.1. Methodological Considerations

We acknowledge that the cohort sizes used in the present study are modest. However, such sample sizes are not unusual in the planarian behavioral literature, where experiments frequently rely on either small groups of worms observed collectively or limited numbers of individually tracked animals. For example, Paskin et al. used 10 groups of 6 worms per condition to study wavelength-dependent phototactic behavior [39]. Talbot and Schötz reported locomotor analyses using *n* = 5 worms for intra-worm variability and n=15 for broader characterization of wild-type locomotion [40]. Similarly, Pagán et al. quantified seizure-like responses using group sizes ranging from approximately 4–14 worms depending on the assay [41]. More broadly, the planarian model has been widely adopted because it is experimentally tractable, low cost, and easy to maintain [42].

Accordingly, the sample sizes used here are consistent with prior work in this model system rather than unusually small by planarian research standards [39,40,41,42]. Although the cohort size was modest, the interpretability of the present data is strengthened by the observed effect sizes. Statistical power is influenced not only by sample size but also by the magnitude of the underlying effect, such that moderate-to-large effects support the sensitivity of the design to detect biologically meaningful differences. In this context, effect sizes in the medium-to-large range suggest that the principal findings are unlikely to reflect sampling variability alone. Thus, while small or nonsignificant effects should be interpreted cautiously, the observed effect sizes support the view that the study was sufficiently sensitive to detect the main effects of interest.

### 4.2. Acquisition of Reward-Associated Place Preference

The present study demonstrates that planaria can acquire a robust conditioned place preference when sucrose is repeatedly paired with an initially non-preferred surface. Untrained animals displayed a natural bias toward the rough surface, whereas trained animals reversed this preference after repeated association of sucrose with the smooth surface. This shift indicates that the smooth surface acquired positive motivational value through associative learning and further supports the utility of planaria as a tractable model for studying reward-related behavior.

This finding is in line with earlier work showing that sucrose can function as a rewarding stimulus in planaria and can induce addiction-like behavioral responses [6,23]. In the present study, however, the significance of the intact-animal CPP extends beyond simply reproducing prior observations. Establishing a clear and reproducible conditioned preference in intact animals was essential because the central aim of the study was to determine whether a previously acquired reward-associated behavioral state could remain detectable after amputation and regeneration. The intact CPP results therefore serve as the necessary conceptual and experimental foundation for interpreting all subsequent findings.

More broadly, the successful induction of CPP in this paradigm suggests that reward learning in planaria can be sufficiently stable to serve as a meaningful probe of memory persistence. This point is important because not every behavioral change necessarily reflects a consolidated learned state. A transient motivational bias would provide only limited insight into regeneration-related retention. By contrast, the sustained reversal of the innate surface preference observed here indicates that the conditioned state was strong enough to justify examining whether it could later survive tissue loss and re-emerge in regenerated animals.

### 4.3. Reward Exposure as a Determinant of CPP Formation

The present findings further indicate that the establishment of conditioned place preference depends not only on the availability of reward, but also on the manner in which that reward is delivered. Although sucrose clearly acts as a reinforcing stimulus, prolonged exposure did not reliably generate conditioned preference in the initial training paradigm, whereas shorter and more discrete daily exposures produced a stable CPP. This suggests that reward-driven learning in planaria is shaped not simply by reward magnitude, but by the temporal structure of reward presentation.

One possible interpretation is that prolonged exposure diminishes the salience of the conditioned context or introduces competing physiological effects that weaken the association between the environment and the rewarding stimulus. By contrast, shorter exposures may preserve the distinctiveness of the learning episode and thereby facilitate stronger contextual encoding. In this sense, the results suggest that effective conditioning in planaria depends on a balance between reward intensity and reward timing, a principle that resonates broadly with reinforcement-based learning across model systems.

This issue is especially relevant to the logic of the present study. Because the central question concerned whether a conditioned reward preference could persist through regeneration, it was necessary first to define training conditions capable of producing a robust and reproducible behavioral state. Optimizing the reward exposure paradigm therefore did more than improve conditioning efficiency; it created the essential experimental framework for testing whether a consolidated reward-associated preference could later remain detectable after major anatomical disruption.

### 4.4. Post-Regeneration Persistence of Reward-Associated Memory

The most consequential finding of the present study is that the reward-associated preference established before amputation remained evident after regeneration. This observation provides the clearest evidence that a conditioned behavioral state can survive not only tissue loss due to amputation, but also the extensive anatomical and functional reorganization that accompanies regeneration. The conditioned preference remained evident across both stages: it was detectable as an early behavioral bias immediately after amputation and was subsequently re-expressed after regeneration in both anterior- and posterior-derived animals.

This post-regeneration persistence is central to the broader interpretation of the study because it directly addresses a long-standing question in planarian memory research: whether previously acquired behavioral information can remain accessible after the organism has undergone substantial physical reconstruction. The present findings indicate that sucrose conditioning leaves behind a durable trace that continues to influence behavior after regeneration is complete. From a behavioral standpoint, this suggests that the information underlying reward-associated learning is sufficiently stable to outlast the loss of major body structures and to shape the phenotype of regenerated animals.

Particularly striking is the persistence of the conditioned preference in tail-derived regenerates. Because posterior fragments initially lack the original cephalic ganglion, their later expression of the learned sucrose-associated preference argues strongly against the view that reward memory is stored exclusively in the head. Although the present data do not identify the precise biological substrate of this persistence, they provide compelling evidence that the ability to re-express the conditioned behavior is not solely dependent on continuous preservation of the original brain. This is the central conceptual advance of the study and the reason the post-regeneration findings carry the greatest interpretive weight.

These results extend the broader literature on memory retention in planaria by showing that reward-associated learning, and not only simple environmental conditioning, can remain behaviorally accessible after regeneration [4,27,43]. This is especially important because sucrose-induced CPP is widely regarded as a proxy for addiction-like reward behavior in this model. The present findings therefore suggest that motivationally relevant learned states may persist across profound biological disruption, highlighting planaria as a particularly informative system for studying how reward memories are preserved, transformed, or re-expressed during regeneration.

### 4.5. Implications for Distributed Memory Storage

The persistence of conditioned preference in regenerated animals has important implications for how memory may be organized in planaria. If animals regenerated from posterior fragments can later express a reward-associated preference acquired before amputation, then the relevant memory-related information cannot be assumed to reside exclusively within the original cephalic ganglion. Rather, the findings support the broader possibility that information necessary for behavioral re-expression is distributed across the organism or can be preserved in forms that do not depend on uninterrupted maintenance of the original brain architecture.

This interpretation is consistent with increasing interest in distributed and non-canonical mechanisms of memory storage in regenerative systems [9,13,44]. Several possibilities could, in principle, account for the present findings. Memory-related information may persist within peripheral neural structures such as longitudinal nerve cords and associated sensory circuitry. Alternatively, it may be retained through stable molecular or epigenetic states in non-neural tissues, RNA-mediated signaling pathways, or regeneration-associated programs capable of reconstructing previously acquired behavioral tendencies. Although the present study does not discriminate among these alternatives, it provides a strong behavioral basis for taking such mechanisms seriously.

The immediate post-amputation convergence dynamics provide an important complementary perspective on the retention of reward-associated behavior. Rather than merely influencing the eventual distribution of fragments across surfaces, prior sucrose conditioning appeared to shape the trajectory by which anterior and posterior fragments reoriented immediately after amputation. Fragments derived from conditioned planaria converged more readily toward the reward-associated smooth surface, whereas those from untrained animals continued to exhibit behavior more consistent with the innate rough-surface bias. This pattern is conceptually significant because it suggests that the influence of prior learning remained detectable not only after regeneration, but already during the acute phase following tissue disruption. In this view, conditioning did not simply alter the final behavioral outcome; it appeared to modulate the early directional dynamics of surface selection immediately after amputation. Although the post-regeneration phenotype provides the strongest evidence for persistence of reward-associated memory, these convergence-rate findings imply that the behavioral trace of prior conditioning may remain accessible across successive stages of biological disruption and recovery. Considered together, the immediate post-amputation and post-regeneration observations strengthen the broader interpretation that reward-associated memory in planaria is supported by mechanisms that are not exclusively dependent on the intact cephalic ganglion.

More broadly, these findings carry conceptual significance because sucrose engages dopaminergic and opioid signaling pathways in a manner analogous to addictive substances in vertebrates [6,19,25,45,46]. The ability of a sucrose-associated behavioral state to persist through regeneration suggests that addiction-like reward memories may be encoded in a biologically robust manner that is not readily erased by structural disruption. In this respect, planaria offer a uniquely tractable framework for studying how reward-related internal states are maintained across major anatomical change and may help illuminate broader principles of memory organization that extend beyond this model.

### 4.6. Dopaminergic Regulation of Reward Learning Across Regeneration

The antagonist experiments provide an important mechanistic complement to the regeneration findings by showing that dopaminergic signaling is required for establishment of the sucrose-associated behavioral state that later persists after regeneration. When a D1 dopamine antagonist was present during conditioning, planaria failed to acquire the sucrose-associated preference and instead retained their natural surface bias. This indicates that D1-mediated dopaminergic reinforcement is necessary for the reward-paired context to acquire motivational significance in this paradigm, consistent with previous work implicating dopamine in CPP-like behavior and addiction-related responses in planaria [6,19,23,25].

The regenerated antagonist-treated animals are particularly informative because they largely preserved the innate rough-surface preference rather than the learned smooth-surface preference observed in the sucrose-trained regenerated group. This contrast is important because it indicates that the post-regeneration phenotype is shaped by the learning conditions present during the original conditioning phase rather than emerging as a nonspecific consequence of regeneration itself. Regeneration alone did not generate an arbitrary or stereotyped behavioral outcome; instead, the regenerated phenotype reflected whether dopaminergic reinforcement had been available at the time the reward association was formed.

This finding strengthens the interpretation that D1-dependent signaling is required for establishing conditioned reward preference that is subsequently preserved and re-expressed after regeneration. If the reward-associated state fails to form under dopamine receptor blockade, then there is no conditioned phenotype to be re-expressed following tissue reconstruction. In this sense, the antagonist experiments show not only that dopamine is important for immediate reward learning, but also that it is necessary for establishing the kind of long-lasting internal state that can subsequently survive major biological disruption.

The fact that this blockade of conditioned preference remained evident after regeneration further sharpens the interpretation of the study. The persistence of the innate preference in antagonist-treated regenerates suggests that dopaminergic signaling is required for creating the reward-associated trace that is later preserved across regeneration. Thus, the antagonist findings do more than replicate prior work on dopamine and CPP; they directly link dopaminergic reinforcement to the enduring expression of reward-associated behavior in a regenerative context. This makes D1-mediated signaling a particularly relevant target for future investigation of how addiction-like behavioral states are encoded, stabilized, and later re-expressed.

### 4.7. Limitations and Future Directions

Despite the strength of the post-regeneration findings, several limitations warrant consideration. First, although the persistence of conditioned preference in tail-derived regenerates argues against memory storage being confined exclusively to the cephalic ganglion, the present behavioral data do not identify the biological substrate responsible for this persistence. The observed retention could reflect the continued influence of peripheral neural structures, molecular or epigenetic states in non-neural tissues, RNA-mediated signaling, neoblast-associated processes, or regeneration-dependent re-establishment of a previously acquired behavioral program. Because the study relied on behavioral readouts alone, it cannot distinguish among these mechanistic possibilities.

Second, the immediate post-amputation experiments require careful interpretation. Fragments in the immediate aftermath of amputation remain in a disrupted physiological state, and their behavior may therefore reflect acute injury responses, stress-related effects, residual neural activity, or early regenerative signaling in addition to any memory-related process. For this reason, the immediate fragment data should be regarded as supportive rather than definitive, whereas the regenerated-animal experiments provide the strongest basis for concluding that reward-associated behavior can persist across regeneration. The major interpretive emphasis of the present study therefore rests on the post-regeneration phenotype rather than on the immediate post-amputation observations.

Third, the behavioral paradigms differed across experiments. The fragment-switching assays were quantified at the group level, whereas the intact-animal CPP assays were assessed at the individual level. These paradigms capture different dimensions of behavior and are therefore not directly quantitatively interchangeable. As a result, comparisons across intact, immediate post-amputation, and regenerated states should be interpreted primarily at the conceptual rather than strictly numerical level. Although the overall behavioral patterns are mutually informative, they do not establish one-to-one equivalence across all experimental contexts.

The modest sample size also remains an important limitation. Although the observed behavioral patterns were coherent and biologically interpretable, larger cohorts would strengthen confidence in the generalizability of these findings and improve precision in estimating the robustness of the effects. In addition, while the antagonist results strongly support disruption of reward learning, the present design does not fully exclude the possibility that dopaminergic blockade may also influence movement-related or exploratory behaviors that secondarily affect surface occupancy. Future experiments incorporating more refined locomotor analysis will therefore be useful in distinguishing specific effects on reward learning from broader behavioral consequences.

Future studies should examine memory retention across multiple post-amputation time points in order to better separate acute injury-related effects from stable post-regeneration behavioral expression. Automated tracking and higher-resolution behavioral analysis would further improve sensitivity and reduce potential observer bias. At the mechanistic level, molecular and cellular follow-up studies—including transcriptomic, epigenetic, and fragment-specific profiling approaches—will be required to determine how reward-associated information is preserved and later re-expressed after regeneration [10,11,12,44,47]. It will also be important to investigate the contribution of additional dopaminergic receptors and other neurotransmitter systems to the acquisition, maintenance, and disruption of reward-associated memory [19,25]. Collectively, such work may clarify how addiction-like behavioral states are encoded and sustained in regenerative systems, with broader relevance to general principles of memory and reward biology [20,21,43].

## 5. Conclusions

The present study demonstrates that sucrose can induce a robust conditioned place preference in planaria and, more importantly, that this reward-associated behavioral state can persist after amputation and subsequent regeneration. Both immediate post-amputation observations and post-regeneration behavioral testing support the interpretation that the learned sucrose-associated preference is not dependent exclusively on the continuous presence of the original cephalic ganglion. The retention and later re-expression of conditioned preference in animals regenerated from posterior fragments therefore provide behavioral evidence consistent with distributed or non-cephalic mechanisms of memory storage. These findings extend the planarian memory literature by showing that reward-associated learning, including an addiction-like behavioral phenotype, can remain detectable despite major anatomical disruption and reconstruction.

The study further shows that dopaminergic signaling is essential for the formation of this persistent reward-associated state. Blocking D1 dopamine receptors during training prevented the acquisition of sucrose-induced conditioned place preference and abolished its later expression after regeneration, indicating that dopamine-dependent reinforcement is required not only for immediate reward learning but also for establishing the enduring trace that survives regenerative remodeling. Taken together, these results highlight planaria as a powerful tool for investigating how reward memories are encoded, stabilized, and re-expressed outside conventional centralized brain frameworks. More broadly, the findings provide a foundation for future mechanistic studies aimed at identifying the molecular, cellular, and neural substrates that support distributed memory storage in regenerative organisms.

## Figures and Tables

**Table 1:** Fisher’s exact test comparing surface preference of sucrose-trained and untrained amputated planaria segments.

Time (min)	Untrained Smooth	Untrained Rough	Trained Smooth	Trained Rough	*p*-Value
3	17	3	10	20	0.0004
15	7	13	22	8	0.0096
30	4	16	27	3	$6.634\times 10^{-7}$

**Table 2:** Two-factor RM ANOVA of time spent by sucrose-trained regenerated planaria on different surfaces.

Source	SS	df	MS	*F*	$\eta_{p}^{2}$
(Intercept)	$1.944\times 10^{7}$	1	$1.944\times 10^{7}$	$-5.34\times 10^{19}$	0.972
Error	$-1.819\times 10^{-12}$	5	$-3.638\times 10^{-13}$	–	−0.000
Region (Head vs. Tail)	$7.755\times 10^{-26}$	1	$7.755\times 10^{-26}$	$-2.13\times 10^{-13}$	0.000
Surface (Smooth vs. Rough)	$1.694\times 10^{6}$	1	$1.694\times 10^{6}$	13.45	0.749
Region × Surface	$7.086\times 10^{5}$	1	$7.086\times 10^{5}$	6.245	0.555
Error (Surface)	$6.297\times 10^{5}$	5	$1.259\times 10^{5}$	–	0.526
Error (Region:Surface)	$5.673\times 10^{5}$	5	$1.135\times 10^{5}$	–	0.500

**Table 3:** Post-hoc paired *t*-tests (BH-corrected) comparing time spent on the smooth vs. rough surface.

Comparison	*t*	*p*	$p_{\mathrm{BH}}$	Cohen’s *d*
Head Smooth vs. Head Rough	2.643	0.0458	0.0458	1.079
Tail Smooth vs. Tail Rough	3.200	0.0240	0.0480	1.306

**Table 4:** Nonparametric two-factor RM analysis of sucrose+antagonist-trained regenerated planaria surface preference.

Factor	$\eta_{H}^{2}$	*p*	Post-Hoc Comparison	$r_{\mathrm{rb}}$ ($p_{\mathrm{BH}}$)
Region (Head vs. Tail)	0.000	0.6109	–	–
Surface (Smooth vs. Rough)	1.000	0.0011	Head: Smooth vs. Rough	0.899 (0.0625)
			Tail: Smooth vs. Rough	0.899 (0.0312)
Region × Surface	0.000	0.6109	–	–

**Figure A1:**
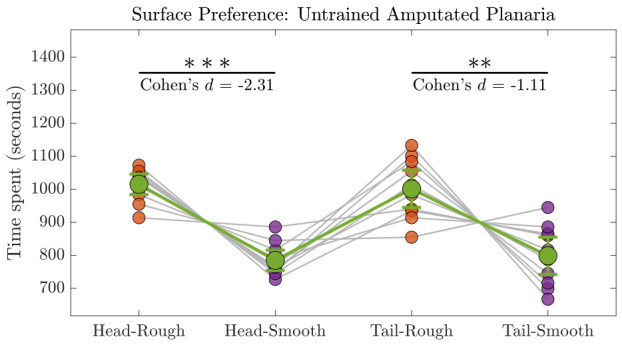
Two-factor RM ANOVA analysis of time spent by untrained amputated planaria segments (n=10) on different surfaces right after amputation. Individual gray lines denote the trajectories of individual planaria across the four conditions: Head–Smooth, Head–Rough, Tail–Smooth, and Tail–Rough. Green dots indicate the mean time spent in each condition, with 95% confidence intervals shown as error bars. Green lines connect the means to highlight the overall trajectory across conditions. Post-hoc paired *t*-tests revealed that the untrained head ($p_{\mathrm{BH}}=1.0\times 10^{-4},\;d=-2.31$) and tail ($p_{\mathrm{BH}}=0.0065,\;d=-1.11$) regions spent significantly more time on the rough surface compared to the smooth surface. Note that a significance at p<0.01 and p<0.001 are denoted by ∗∗ and ∗∗∗, respectively.

**Figure A2:**
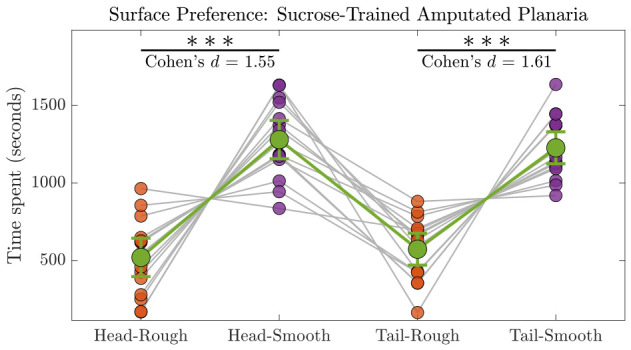
Two-factor RM analysis of time spent by sucrose-trained amputated planaria segments (n=15) on the rough and smooth surfaces right after amputation. Individual gray lines trace the trajectories of individual planaria across the four conditions: Head–Smooth, Head–Rough, Tail–Smooth, and Tail–Rough. Green dots represent the mean time spent in each condition, with error bars indicating 95% confidence intervals. Green lines connect the condition means to emphasize the overall pattern across conditions. Post-hoc paired *t*-tests revealed that the sucrose-trained head ($p_{\mathrm{BH}}<0.0001,\;d=1.55$) and tail ($p_{\mathrm{BH}}<0.0001,\;d=1.61$) segments spent significantly more time on the smooth surface compared to the rough surface, thus ensuring the retention of reward-associated memory. Note that a significance at p<0.001 is denoted by ∗∗∗.

## Data Availability

The data collected during this project has been uploaded to the following link: https://doi.org/10.6084/m9.figshare.31337869.

## Abbreviations

The following abbreviations are used in this manuscript:

CPP	conditioned place preference
RNA	ribonucleic acid
P+W	preferred surface paired with water
U+S	unpreferred surface paired with 10% sucrose solution
U+S+D	unpreferred surface paired with 10% sucrose solution and D1 dopamine antagonist
SEM	standard error of the mean
BH	Benjamini–Hochberg
RMSE	root mean square error
CI	confidence interval
RM	repeated measures
ANOVA	analysis of variance
ANCOVA	analysis of covariance

## Appendix A. Surface Preference of Amputated Planaria

### Appendix A.1. Untrained Planaria

Two-Factor RM ANOVA

After ensuring the normality of data by Shapiro–Wilk test, a two-factor RM ANOVA was conducted to examine the effects of Region and Surface on the measured outcome (Figure A1). The main effect of Region was not significant, F(1,9)=0.00, p=1.00, $\eta_{p}^{2}=0.00$. In contrast, there was a significant main effect of Surface, F(1,9)=32.46, $p=2.95\times 10^{-4}$, $\eta_{p}^{2}=0.88$, indicating a strong influence of surface type. The Region × Surface interaction was not significant, F(1,9)=0.27, p=0.617, $\eta_{p}^{2}=0.03$.

Post-Hoc Comparisons

Post-hoc paired *t*-tests with BH correction revealed significant differences between smooth and rough surfaces in both regions. For the head region, subjects spent significantly less time on the smooth surface compared to the rough surface, t(9)=−7.30, $p_{\mathrm{BH}}=1.0\times 10^{-4}$, d=−2.31. Similarly, for the tail region, a significant difference was observed, t(9)=−3.52, $p_{\mathrm{BH}}=0.0065$, d=−1.11.

### Appendix A.2. Sucrose-Trained Planaria

Two-Factor RM ANOVA

A two-factor RM ANOVA was conducted to examine the influence of Region and Surface on the observed behavior (Figure A2) after confirming normality using the Shapiro–Wilk test. The main effect of Region was not significant, $F\left(1,14\right)=-2.49\times 10^{-13}$, p=1.00, $\eta_{p}^{2}\approx 0.00$, suggesting no detectable difference between regions. In contrast, Surface significantly affected the outcome, F(1,14)=66.81, $p=1.07\times 10^{-6}$, $\eta_{p}^{2}=0.86$, indicating a marked difference between surface types. The Region × Surface interaction was not significant, F(1,14)=0.47, p=0.506, $\eta_{p}^{2}=0.03$.

Post-Hoc Comparisons

Post-hoc paired *t*-tests with BH correction confirmed significant differences between smooth and rough surfaces for both regions. For the head region, t(14)=6.02, $p_{\mathrm{BH}}<0.0001$, d=1.55, and for the tail region, t(14)=6.22, $p_{\mathrm{BH}}<0.0001$, d=1.61, both indicating large effect sizes.

Results from two-factor RM ANOVA and post-hoc analyses are summarized in Table A1.

**Table A1:** Summary of two-factor RM ANOVAs for two datasets. Partial eta-squared ($\eta_{p}^{2}$) is reported for effect sizes.

Effect	DF	F	*p*-Value	$\eta_{p}^{2}$
**Region**	1, 9/1, 14	0.00/0.00	1.00/1.00	0.00/0.00
**Surface**	1, 9/1, 14	32.46/66.81	$2.95\times 10^{-4}$/$1.07\times 10^{-6}$	0.88/0.86
**Region × Surface**	1, 9/1, 14	0.27/0.47	0.617/0.506	0.03/0.03
**Post-hoc paired *t*-tests (BH-corrected)**				
Dataset 1 Head	t(9)=−7.30, $p_{\mathrm{BH}}=1.0\times 10^{-4}$, d=−2.31			
Dataset 1 Tail	t(9)=−3.52, $p_{\mathrm{BH}}=0.0065$, d=−1.11			
Dataset 2 Head	t(14)=6.02, $p_{\mathrm{BH}}<0.0001$, d=1.55			
Dataset 2 Tail	t(14)=6.22, $p_{\mathrm{BH}}<0.0001$, d=1.61			
